# Supplementary material for: Drought, armed conflict and population mortality in Somalia, 2014–2018: A statistical analysis
Source: PLOS Glob Public Health. 2023 Apr 12;3(4):e0001136. doi: 10.1371/journal.pgph.0001136 (PMC10096495; doi:10.1371/journal.pgph.0001136)
Supplement: S1 Text — (DOCX) [file pgph.0001136.s001.docx]

Drought, armed conflict and population mortality in Somalia, 2014-2018: a statistical analysis

S1 Text: Additional methods and results

Table of contents

[Computation of terms of trade indicators 2](#_Toc130220077)

[Additional tables and figures 3](#_Toc130220078)

[Mortality surveys 3](#_Toc130220079)

[Population denominators and displacement 6](#_Toc130220080)

[Models’ predictive accuracy 9](#_Toc130220081)

[Mortality estimates 11](#_Toc130220082)

[Trends in terms of trade indicators 17](#_Toc130220083)

[Sensitivity analyses 18](#_Toc130220084)

# Computation of terms of trade indicators

FSNAU field researchers collect monthly data from 50 rural and 50 urban markets located throughout Somalia. We extracted price data for the following items: 1 Kg white sorghum; 1 Kg red sorghum; 1 Kg yellow maize; 1 Kg white maize; 1 Kg imported red rice; 1 Kg wheat flour; 1 goat of local quality (i.e. not for export); daily wage in the local currency. Sorghum and maize are locally produced while wheat and rice are mostly imported.

We combined the above variables to compute two terms of trade food security indicators for each district-month: (i) the Kcal equivalent of staple cereal that can be purchased by a typical daily wage, and (ii) the Kcal staple cereal equivalent of the selling price of a local quality goat. For any given market, only price items with ≥ 75% data completeness over the period 2013 to 2018 were retained in the analysis.

The equation for the terms of trade (ToT) of a typical daily wage, for a given district-month, is as follows:

$${ToT}_{W,k,t}=\frac{\sum_{m}^{M_{k}} \frac{\sum_{c}^{C_{m}} \left( \frac{W_{m,t}}{I_{c,m,t}}r_{c}E_{c} \right)^{2}}{\sum_{c}^{C_{m}} \frac{W_{m,t}}{I_{c,m,t}}r_{c}E_{c}}U_{m}}{\sum_{m}^{M_{k}} U_{m}}$$

where $k$ is a district, $t$ a month, $m$ one of the $M_{k}$ markets within the district, $c$ one of the $C_{m}$ cereals with sufficient data completeness in market $m$, $I_{c}$ is the price of 1 Kg of cereal $c$, $W_{m,t}$ is the average daily labour wage in market $m$ and month $t$, $r_{c}$ is the proportion of cereal $c$ that is recovered after milling, $E_{c}$ is the Kcal value of 1 Kg of milled cereal $c$ and $U_{m}$ is a weight equal to 3 for urban markets and 1 for rural markets.

We first computed the monthly terms of trade for each cereal-market time series by working out the Kcal equivalent of the amount of that cereal that a daily wage could purchase, based the price of 1 Kg of the cereal, the proportion lost after milling and an assumed Kcal per Kg.

Whenever more than one cereal-market time series was sufficiently complete for analysis (e.g. white sorghum, red rice), we took a weighted mean of these. In the absence of data on relative purchase quantities of different cereals, we assumed based on simple elasticity principles that, in the context of a poor harvest and high poverty levels, people would have had a strong preference for purchasing the cheapest cereal, and would have shifted their preference according to cereal prices, with a greater tendency to shift to cheaper staples, the lower the daily wage. We represented this assumed elasticity by using the ToT for each cereal itself as a weight when averaging all cereals (mathematically this is equivalent to averaging the squares of each cereal’s ToT).

Lastly, if more than one market was located within the district, we calculated a weighted mean of ToT from each market, weighting urban markets three times more than rural ones.

We replicated the above calculation for the ToT of a local-quality goat, by substituting $W_{m,t}$ with $G_{m,t}$, the price of a local-quality goat.

# Additional tables and figures

## Mortality surveys


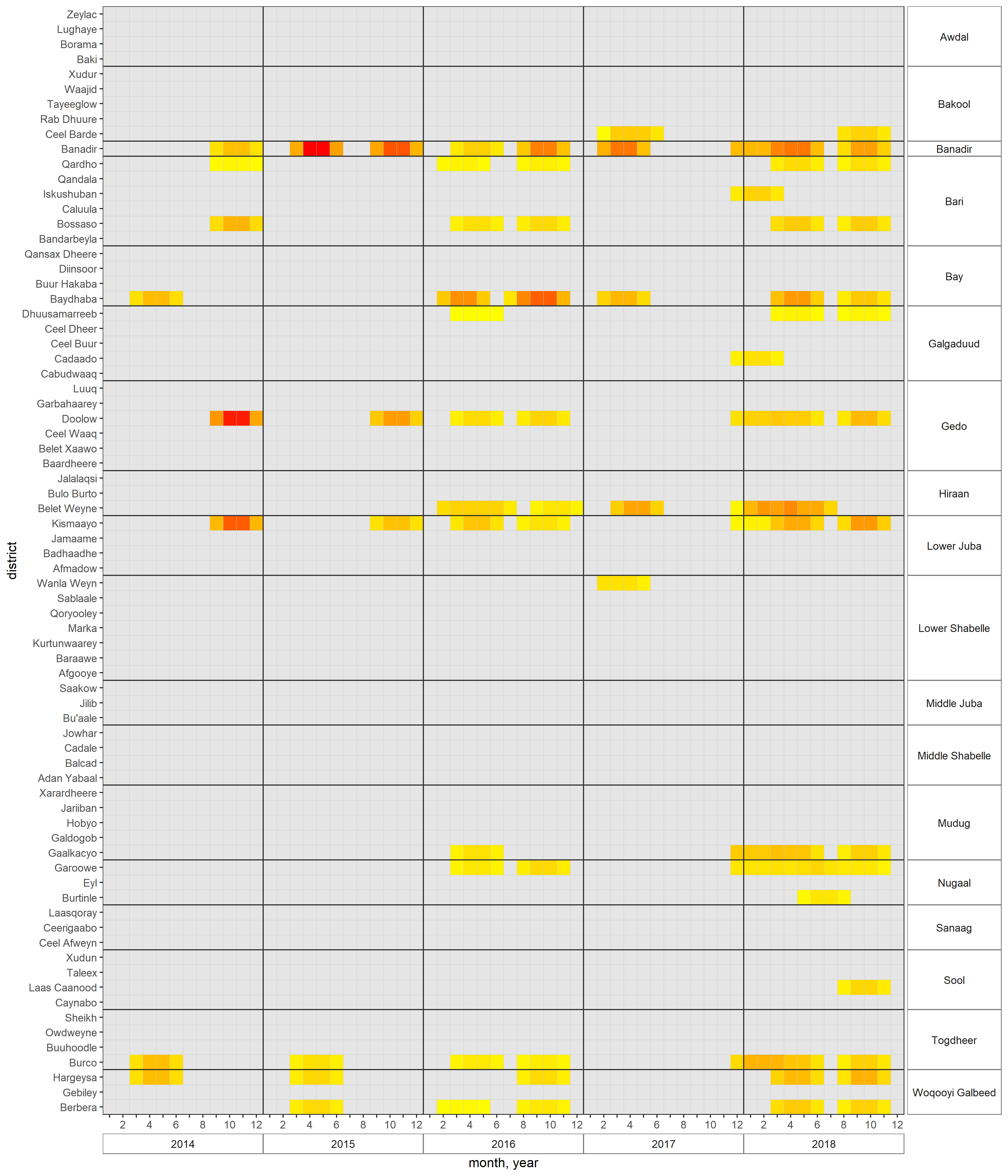


Fig A. Schematic of survey district-month coverage (districts are further grouped by region: see right-hand y axis). Grey indicates that the district-month is not included in any survey’s sampling frame. The intensity of heat colours is proportional to a data availability index for each stratum-month, calculated as follows: (i) first, we multiplied the total survey person-time, quality score and proportion of the month included in the survey’s recall period; (ii) if more than one survey covered the same district-month, we summed the data availability indices of all surveys concerned; (iii) lastly, we rescaled the index to $[0, 1]$ by dividing all values by their maximum.


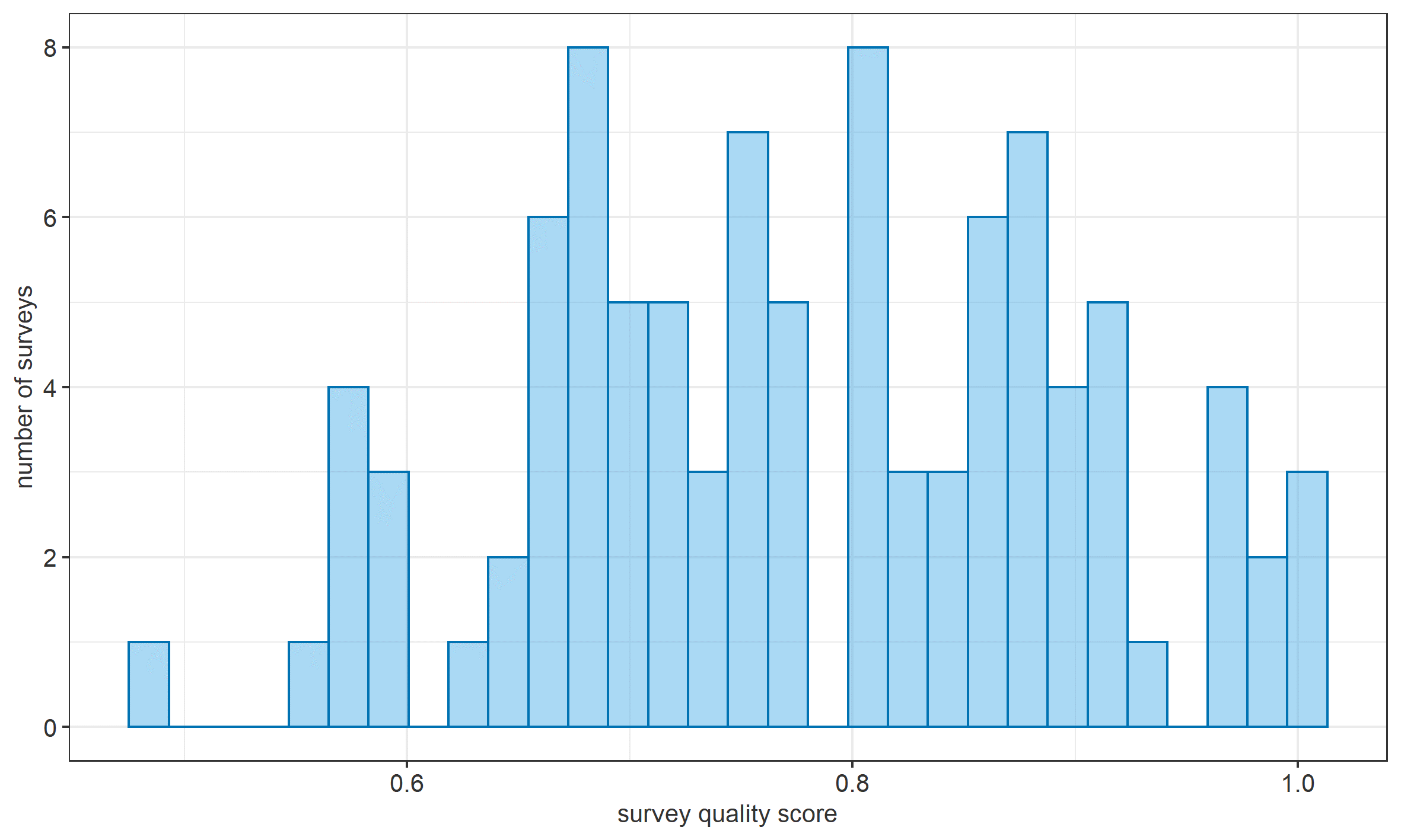


Fig B. Frequency distribution of mortality survey quality score.


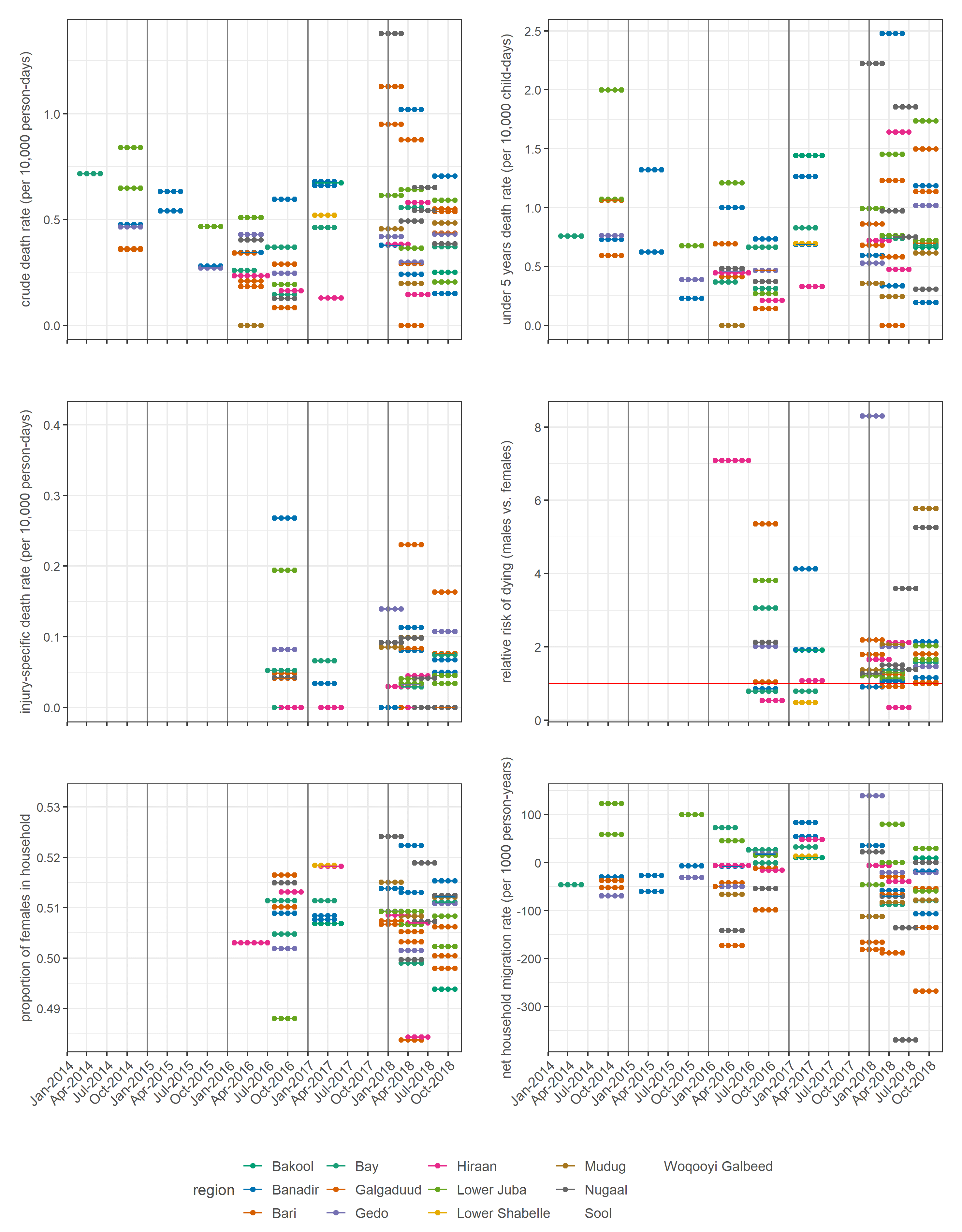


Fig C. Crude trends in key survey-estimated indicators. Each dotted segment represents the point estimate of a survey, with dots being the months falling within the survey’s recall period. Note that there are no observations from Sool and Woqooyi Galbeed regions. Surveys prior to 2016 relied on an ‘aggregate’ questionnaire that did not collect cause of death and gender of individual household members.

## Population denominators and displacement


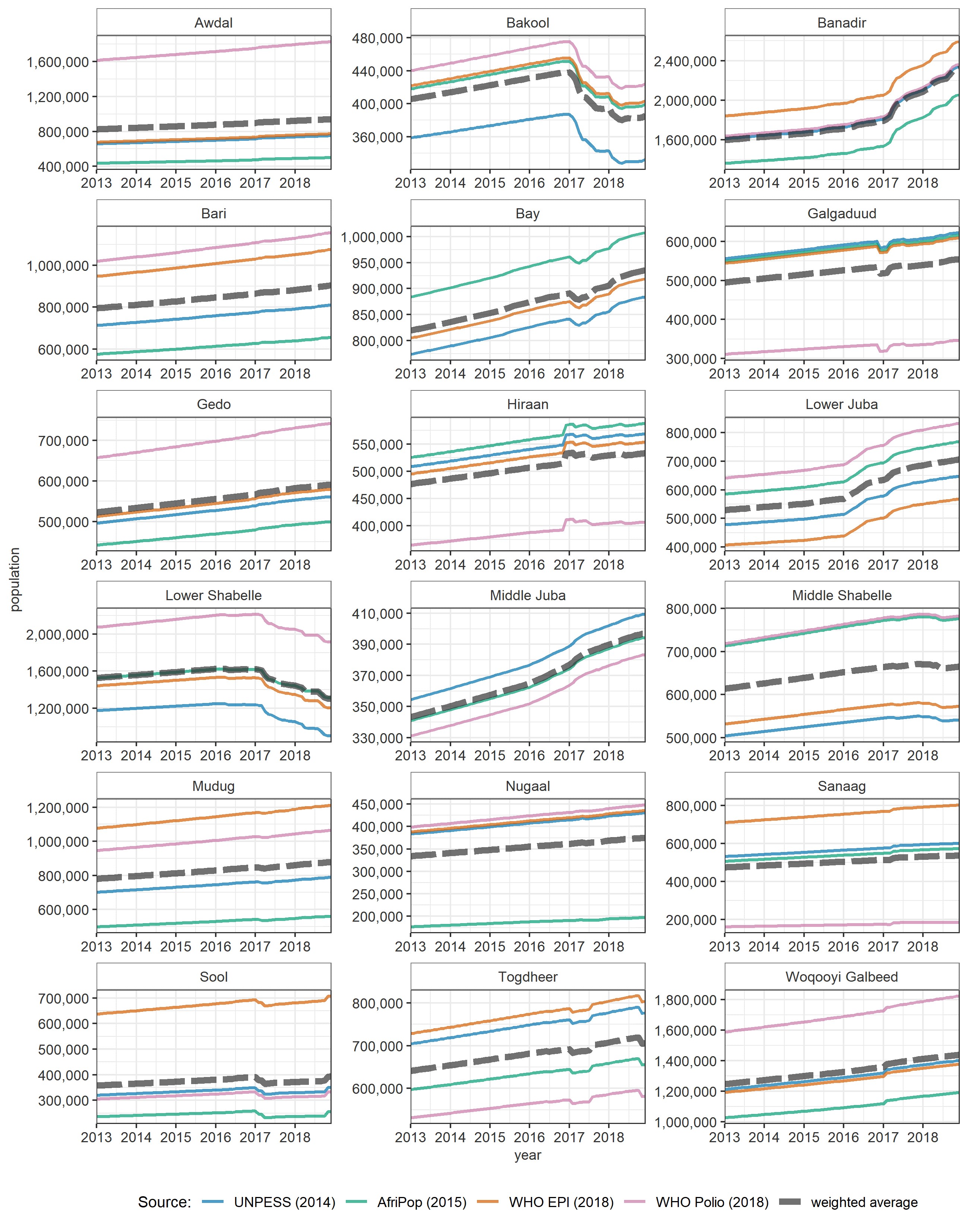


Fig D. Evolution of estimated population denominators, by region and source.


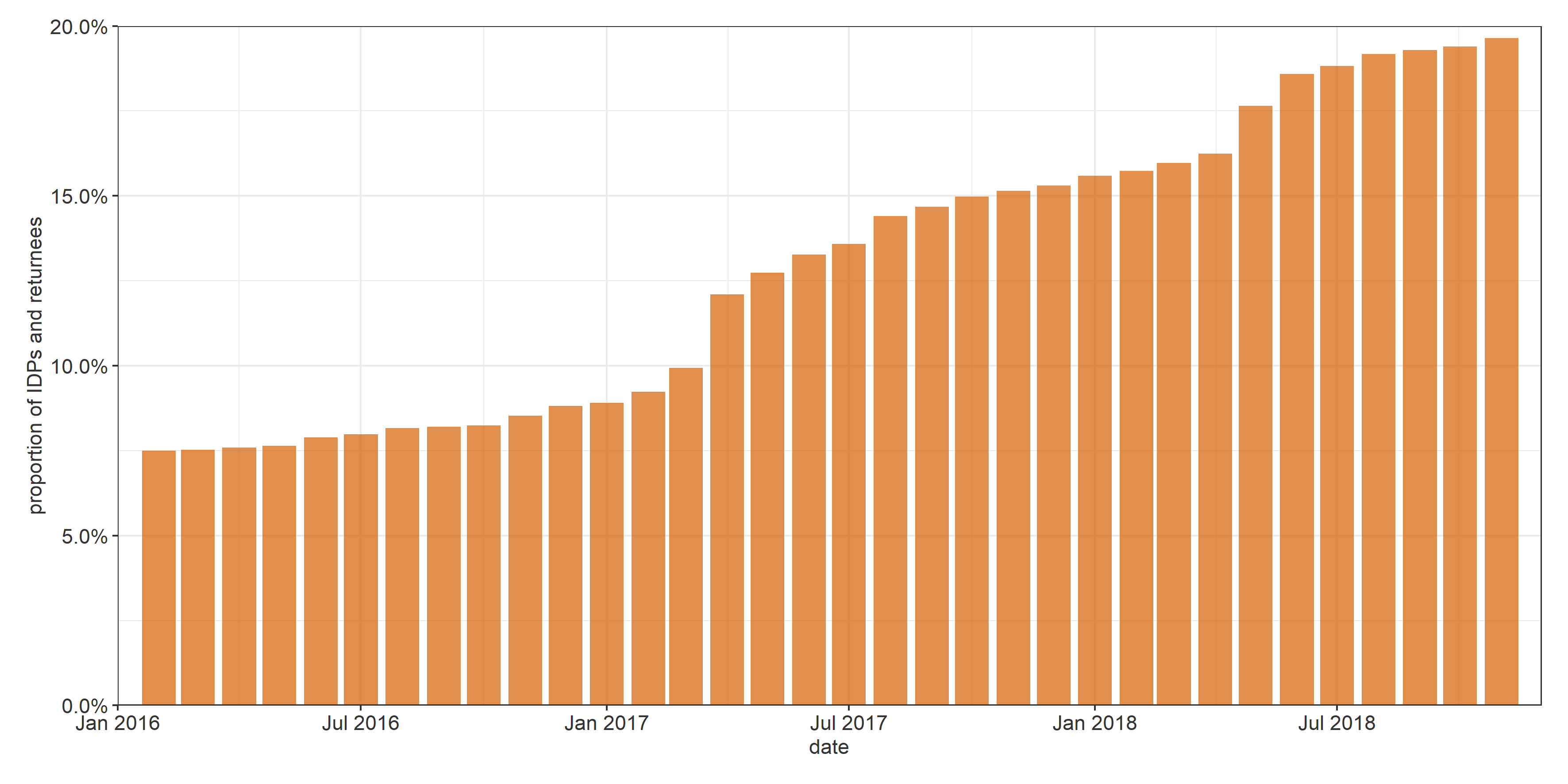


Fig E. Evolution of the estimated proportion of IDPs and returnees across Somalia.


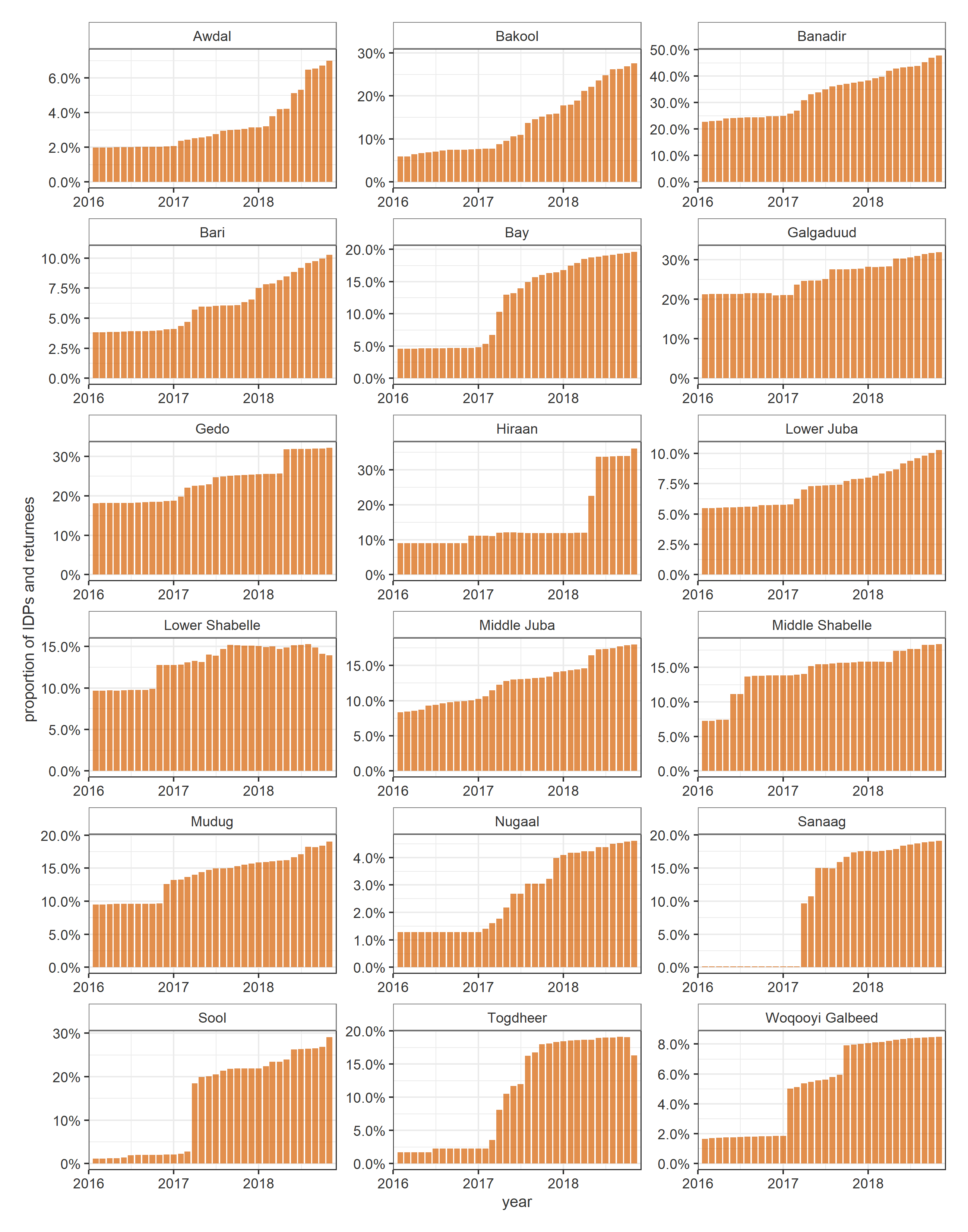


Fig F. Evolution of the proportion of IDPs and returnees, by region.

## Models’ predictive accuracy


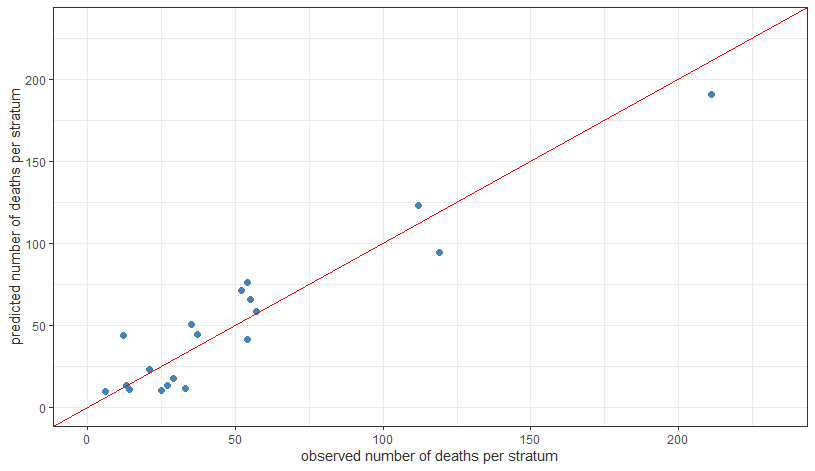


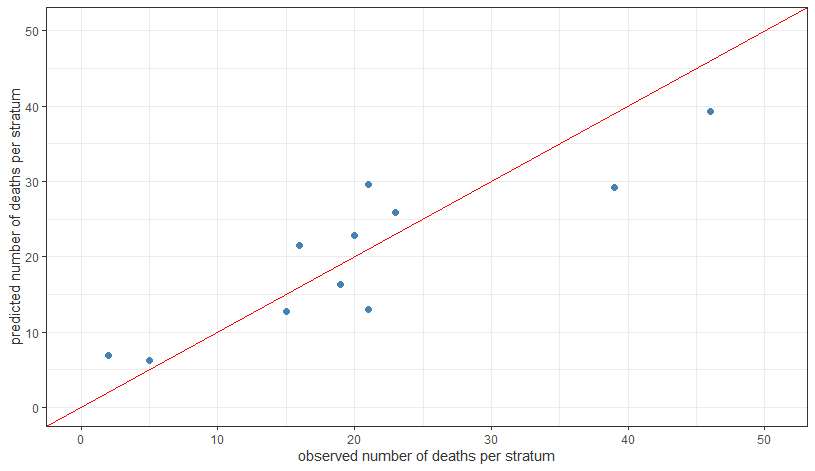


Fig G. Observed versus predicted number of all-age deaths, by stratum (district), after 10-fold cross-validation (top panel) and prediction on the holdout sample (bottom panel). The red line indicates perfect fit.

On 10-fold cross-validation, the model predicted 970 deaths on average, compared to 966 observed across all surveys in the training sample. Corresponding totals for the holdout sample were 227 and 223.


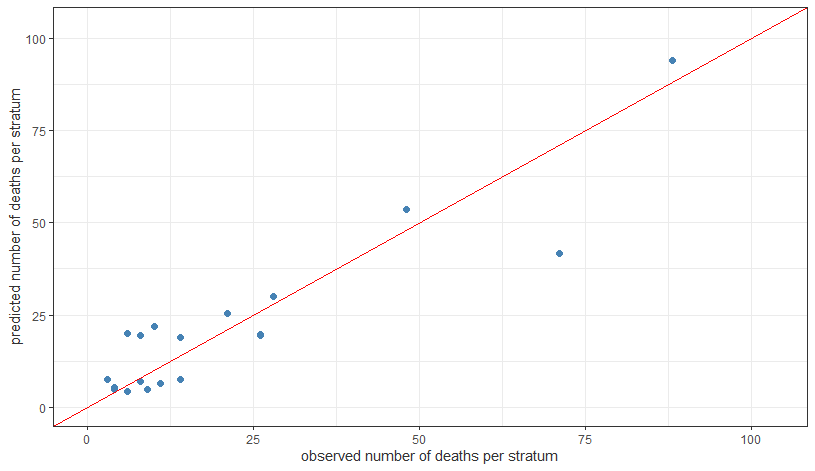


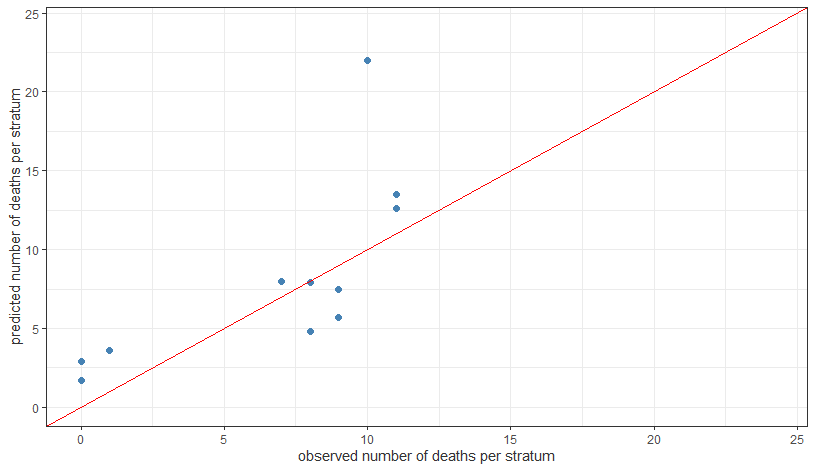


Fig H. Observed versus predicted number of under 5y deaths, by stratum (district), after 10-fold cross-validation (top panel) and prediction on the holdout sample (bottom panel). The red line indicates perfect fit.

On 10-fold cross-validation, the model predicted 412 deaths on average, compared to 405 observed across all surveys in the training sample. Corresponding totals for the holdout sample were 90 and 74.

## Mortality estimates

Table A. Point estimates of crude death rate, excess death rate and excess death toll, under the most likely counterfactual scenario, by district, for all ages and for children under 5y.

| District | Crude death rate | Excess death rate | Excess death toll | Under 5 years death rate | Excess death rate, children under 5y | Excess death toll, children under 5y |
| --- | --- | --- | --- | --- | --- | --- |
| Adan Yabaal | 0.23 | -0.01 | 0 (-100 to 0) | 0.67 | -0.02 | 0 (0 to 0) |
| Afgooye | 0.41 | 0.03 | 800 (-500 to 1300) | 0.88 | -0.03 | -300 (-1200 to 200) |
| Afmadow | 0.71 | 0.15 | 1800 (1400 to 2400) | 1.15 | 0.33 | 1000 (700 to 1600) |
| Baardheere | 0.68 | -0.01 | -100 (-400 to 100) | 1.04 | 0.02 | 100 (-100 to 200) |
| Badhaadhe | 0.47 | 0.01 | 0 (0 to 100) | 0.91 | -0.03 | 0 (-100 to 0) |
| Baki | 0.34 | 0.04 | 200 (100 to 300) | 0.44 | 0.02 | 0 (0 to 100) |
| Balcad | 0.46 | -0.02 | -200 (-600 to 0) | 0.90 | 0.00 | 0 (-300 to 200) |
| Banadir | 0.50 | 0.01 | 1200 (-3700 to 4200) | 0.80 | 0.02 | 900 (800 to 1200) |
| Bandarbeyla | 0.42 | 0.06 | 100 (100 to 200) | 0.48 | 0.01 | 0 (0 to 0) |
| Baraawe | 0.58 | 0.17 | 1100 (900 to 1400) | 1.13 | 0.16 | 300 (200 to 300) |
| Baydhaba | 0.42 | 0.00 | -100 (-1000 to 400) | 0.73 | -0.01 | -100 (-100 to 0) |
| Belet Weyne | 0.44 | 0.09 | 1900 (1700 to 1900) | 0.99 | 0.23 | 1200 (800 to 1200) |
| Belet Xaawo | 0.52 | 0.09 | 600 (300 to 700) | 0.94 | 0.16 | 300 (0 to 300) |
| Berbera | 0.48 | 0.14 | 1600 (1200 to 2400) | 0.65 | -0.02 | -100 (-100 to 0) |
| Borama | 0.35 | -0.04 | -2000 (-2100 to -1500) | 0.41 | -0.05 | -600 (-700 to -200) |
| Bossaso | 0.39 | 0.17 | 5200 (4400 to 5800) | 0.45 | 0.10 | 700 (200 to 800) |
| Bu'aale | 0.40 | 0.08 | 600 (400 to 900) | 0.97 | 0.21 | 400 (200 to 700) |
| Bulo Burto | 0.54 | 0.05 | 600 (300 to 1100) | 1.08 | 0.13 | 400 (100 to 900) |
| Burco | 0.42 | 0.02 | 800 (500 to 1200) | 0.51 | -0.03 | -200 (-300 to -200) |
| Burtinle | 0.53 | 0.21 | 1300 (800 to 2000) | 0.61 | 0.13 | 200 (100 to 400) |
| Buuhoodle | 0.71 | 0.16 | 900 (400 to 1700) | 0.72 | 0.09 | 100 (0 to 300) |
| Buur Hakaba | 0.69 | 0.16 | 1800 (1500 to 2100) | 1.01 | 0.07 | 200 (0 to 200) |
| Cabudwaaq | 0.42 | -0.10 | -600 (-1300 to -300) | 0.81 | -0.22 | -400 (-600 to -200) |
| Cadaado | 0.49 | 0.04 | 300 (200 to 300) | 0.70 | -0.06 | -100 (-200 to -100) |
| Cadale | 0.42 | 0.18 | 1000 (700 to 1400) | 0.83 | 0.18 | 200 (200 to 400) |
| Caluula | 0.43 | 0.12 | 400 (300 to 700) | 0.49 | 0.06 | 100 (0 to 100) |
| Caynabo | 0.51 | 0.17 | 800 (500 to 1200) | 0.56 | 0.11 | 100 (100 to 200) |
| Ceel Afweyn | 0.75 | 0.16 | 1100 (600 to 2000) | 0.73 | 0.10 | 200 (0 to 400) |
| Ceel Barde | 0.41 | 0.07 | 300 (200 to 400) | 0.93 | 0.13 | 100 (100 to 200) |
| Ceel Buur | 0.49 | -0.07 | -600 (-800 to -400) | 0.82 | -0.16 | -300 (-500 to -200) |
| Ceel Dheer | 0.33 | 0.05 | 400 (300 to 500) | 0.59 | 0.11 | 200 (200 to 300) |
| Ceel Waaq | 0.55 | 0.24 | 900 (600 to 1300) | 1.14 | 0.45 | 400 (300 to 600) |
| Ceerigaabo | 0.46 | 0.11 | 1900 (1200 to 2900) | 0.62 | 0.08 | 300 (200 to 700) |
| Dhuusamarreeb | 0.59 | 0.21 | 2300 (1800 to 2600) | 0.99 | 0.32 | 800 (800 to 900) |
| Diinsoor | 0.45 | -0.13 | -1000 (-1400 to -700) | 0.94 | -0.09 | -200 (-200 to -100) |
| Doolow | 0.56 | 0.14 | 600 (500 to 800) | 0.86 | 0.18 | 200 (200 to 300) |
| Eyl | 0.41 | 0.13 | 800 (500 to 1200) | 0.53 | 0.08 | 100 (100 to 200) |
| Gaalkacyo | 0.45 | 0.11 | 3500 (2500 to 5100) | 0.56 | -0.09 | -700 (-800 to -700) |
| Galdogob | 0.37 | 0.11 | 1300 (900 to 2200) | 0.63 | 0.14 | 400 (200 to 800) |
| Garbahaarey | 0.68 | -0.11 | -900 (-1200 to -800) | 0.96 | -0.22 | -500 (-700 to -300) |
| Garoowe | 0.52 | 0.01 | 200 (-300 to 1000) | 0.71 | 0.05 | 200 (-100 to 700) |
| Gebiley | 0.40 | 0.05 | 600 (300 to 1100) | 0.45 | 0.05 | 100 (100 to 200) |
| Hargeysa | 0.29 | 0.00 | -300 (-600 to 400) | 0.43 | 0.00 | 0 (-300 to 700) |
| Hobyo | 0.46 | 0.15 | 1100 (700 to 1700) | 0.65 | 0.17 | 300 (200 to 600) |
| Iskushuban | 0.57 | 0.23 | 1300 (900 to 1900) | 0.58 | 0.15 | 200 (100 to 300) |
| Jalalaqsi | 0.58 | 0.30 | 1900 (1500 to 2500) | 1.02 | 0.39 | 600 (400 to 900) |
| Jamaame | 0.40 | 0.11 | 1200 (800 to 1600) | 0.82 | 0.16 | 500 (400 to 600) |
| Jariiban | 0.38 | 0.14 | 1000 (600 to 1600) | 0.61 | 0.16 | 300 (200 to 500) |
| Jilib | 0.39 | -0.01 | -100 (-400 to 0) | 0.87 | 0.02 | 0 (-200 to 100) |
| Jowhar | 0.34 | 0.05 | 1100 (1100 to 1100) | 0.87 | -0.01 | -100 (-600 to 200) |
| Kismaayo | 0.50 | -0.07 | -1500 (-3000 to -600) | 0.99 | 0.19 | 1000 (900 to 1000) |
| Kurtunwaarey | 0.34 | 0.01 | 100 (-100 to 100) | 0.74 | -0.06 | -200 (-400 to 0) |
| Laas Caanood | 0.52 | -0.01 | -200 (-300 to 0) | 0.66 | -0.11 | -300 (-500 to -200) |
| Laasqoray | 0.35 | -0.01 | -200 (-300 to 200) | 0.43 | -0.01 | 0 (-100 to 100) |
| Lughaye | 0.36 | 0.07 | 400 (200 to 700) | 0.47 | 0.05 | 100 (0 to 100) |
| Luuq | 0.51 | 0.21 | 1500 (1200 to 1800) | 0.92 | 0.33 | 600 (500 to 600) |
| Marka | 0.28 | -0.06 | -1200 (-2300 to -600) | 0.84 | -0.27 | -1400 (-1800 to -1000) |
| Owdweyne | 0.44 | 0.10 | 600 (400 to 1000) | 0.49 | 0.05 | 100 (0 to 100) |
| Qandala | 0.46 | 0.08 | 800 (600 to 1100) | 0.54 | 0.04 | 100 (100 to 100) |
| Qansax Dheere | 0.41 | 0.00 | 0 (-100 to 0) | 0.90 | 0.01 | 0 (-100 to 100) |
| Qardho | 0.51 | -0.04 | -500 (-700 to -400) | 0.54 | -0.09 | -300 (-400 to -200) |
| Qoryooley | 0.26 | 0.01 | 200 (200 to 200) | 0.77 | 0.00 | 0 (-100 to 200) |
| Rab Dhuure | 0.39 | -0.07 | -200 (-600 to -100) | 0.87 | -0.17 | -100 (-300 to 0) |
| Saakow | 0.38 | 0.11 | 1000 (700 to 1500) | 0.96 | 0.28 | 600 (400 to 1100) |
| Sablaale | 0.29 | -0.03 | -100 (-200 to -100) | 0.68 | -0.07 | -100 (-100 to 0) |
| Sheikh | 0.38 | 0.03 | 200 (100 to 300) | 0.49 | 0.03 | 0 (0 to 100) |
| Taleex | 0.48 | 0.15 | 800 (500 to 1200) | 0.55 | 0.10 | 100 (100 to 200) |
| Tayeeglow | 0.27 | 0.06 | 400 (300 to 500) | 0.67 | 0.09 | 100 (0 to 200) |
| Waajid | 0.43 | -0.01 | -100 (-400 to 100) | 0.88 | -0.05 | -100 (-200 to 0) |
| Wanla Weyn | 0.44 | 0.12 | 1900 (1800 to 1900) | 0.89 | 0.17 | 700 (200 to 800) |
| Xarardheere | 0.38 | 0.13 | 700 (500 to 1100) | 0.56 | 0.09 | 100 (100 to 200) |
| Xudun | 0.41 | 0.04 | 200 (100 to 300) | 0.52 | 0.01 | 0 (0 to 0) |
| Xudur | 0.51 | 0.16 | 1400 (1200 to 1500) | 1.00 | 0.23 | 500 (200 to 600) |
| Zeylac | 0.44 | 0.02 | 100 (0 to 300) | 0.46 | 0.01 | 0 (0 to 100) |


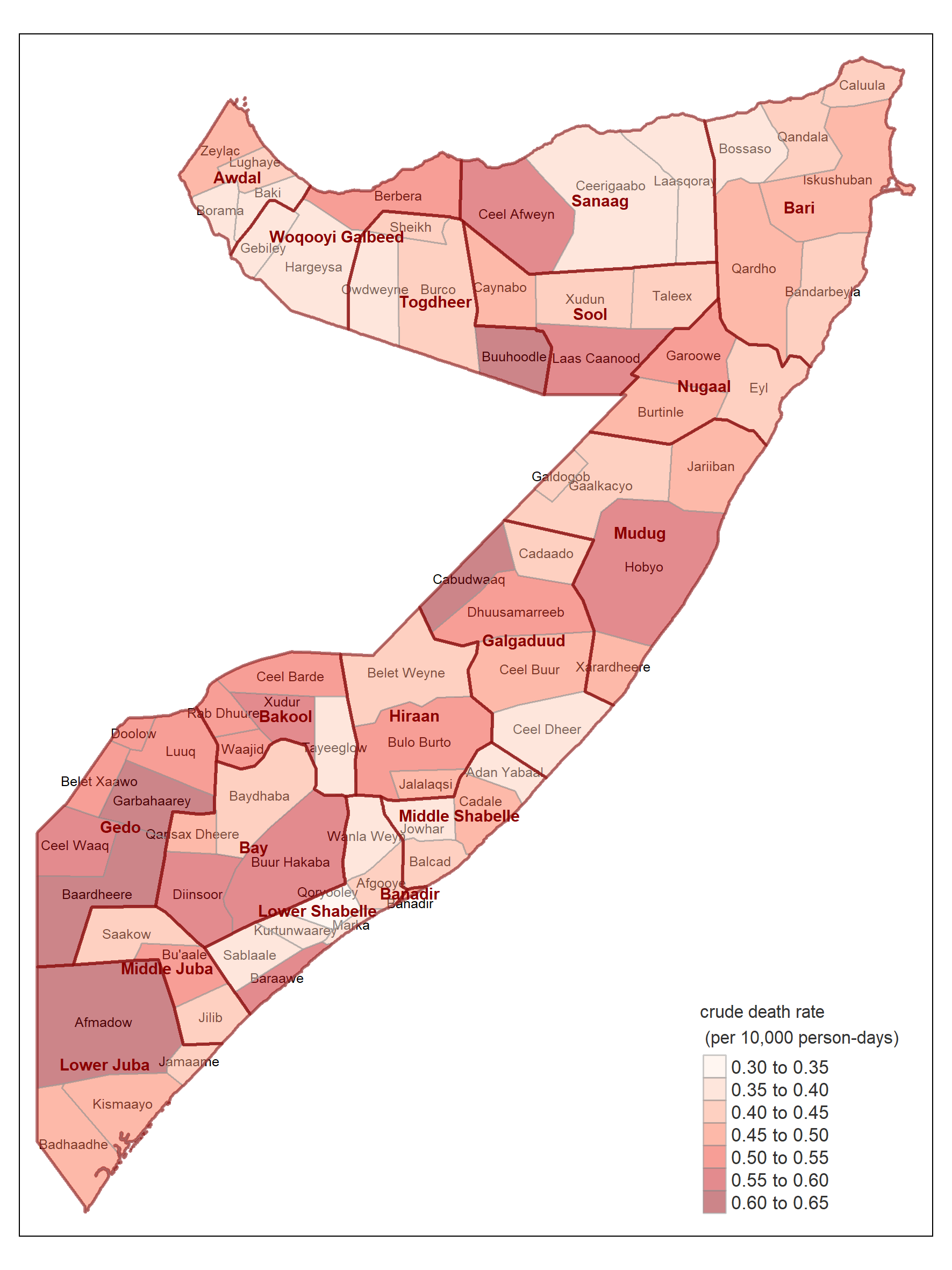


Fig I. Best estimate of crude death rate (CDR), by district. Map created in R software using basemap shapefile provided by the United Nations (<https://data.humdata.org/dataset/cod-ab-som>). Data license: Creative Commons Attribution (CC BY-IGO) (<https://data.humdata.org/faqs/licenses>).


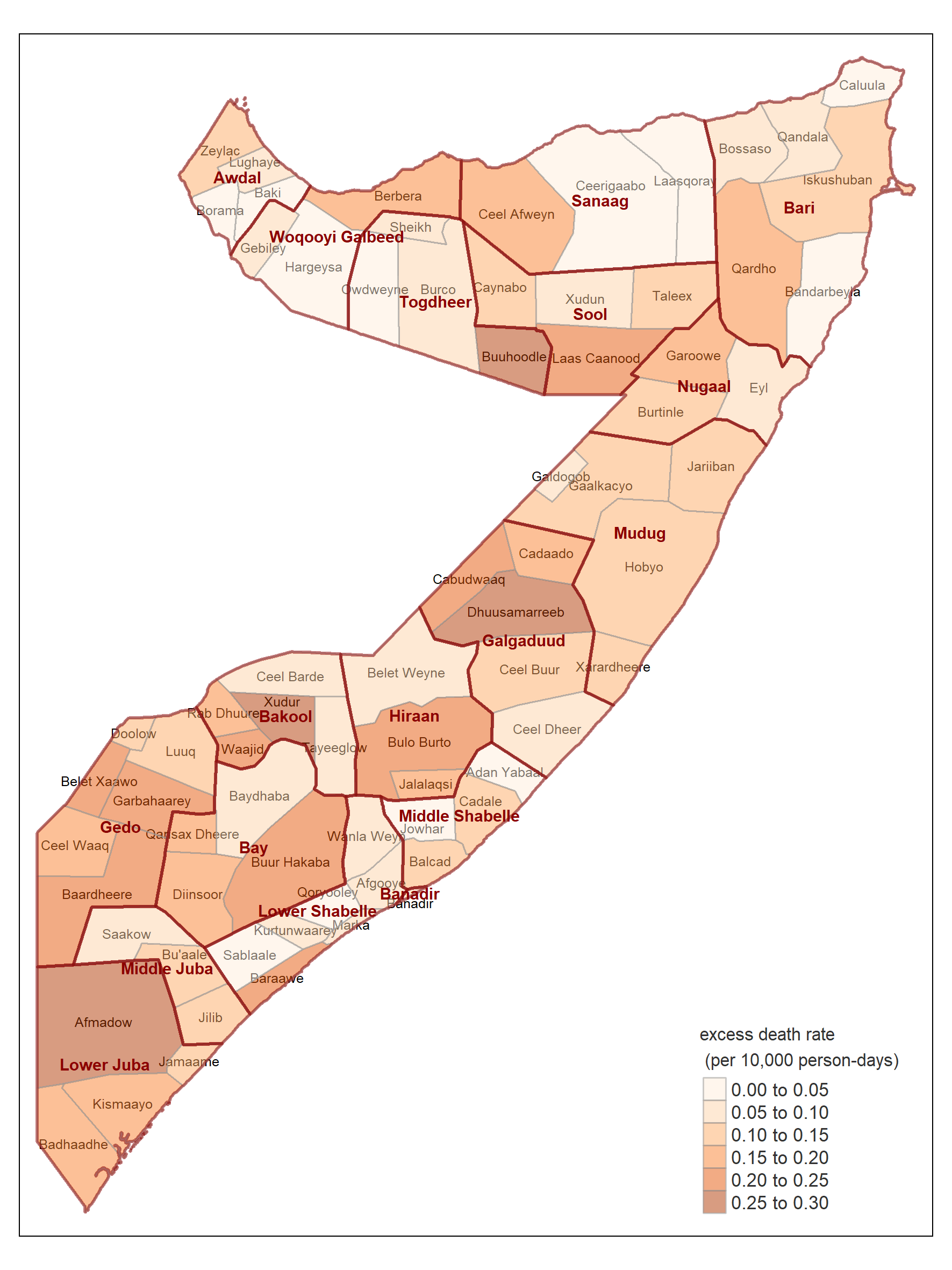


Fig J. Best estimate of excess death rate among all age groups, by district. Map created in R software using basemap shapefile provided by the United Nations (<https://data.humdata.org/dataset/cod-ab-som>). Data license: Creative Commons Attribution (CC BY-IGO) (<https://data.humdata.org/faqs/licenses>).


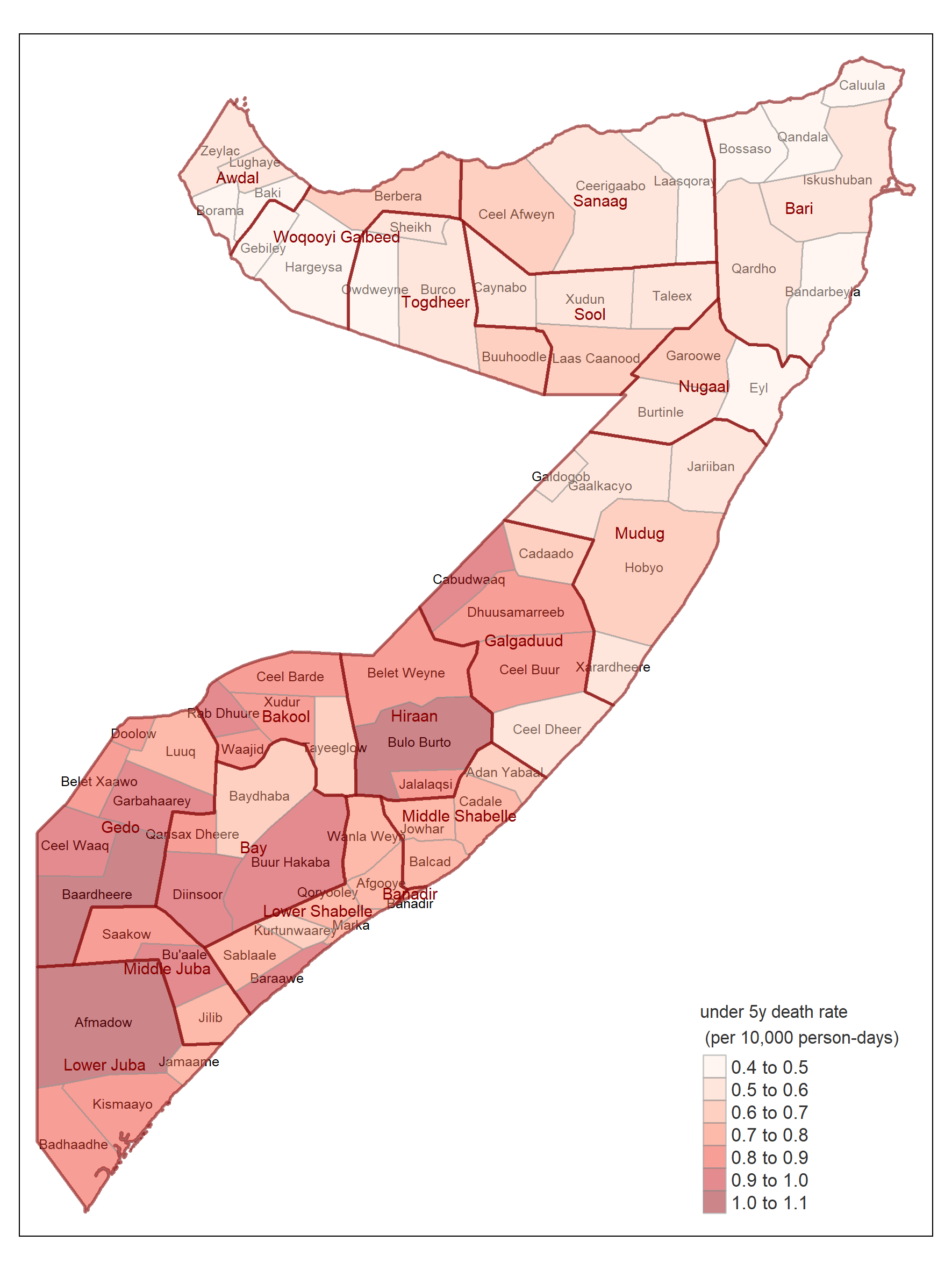


Fig K. Best estimate of under 5 years death rate (U5DR), by district. Map created in R software using basemap shapefile provided by the United Nations (<https://data.humdata.org/dataset/cod-ab-som>). Data license: Creative Commons Attribution (CC BY-IGO) (<https://data.humdata.org/faqs/licenses>).


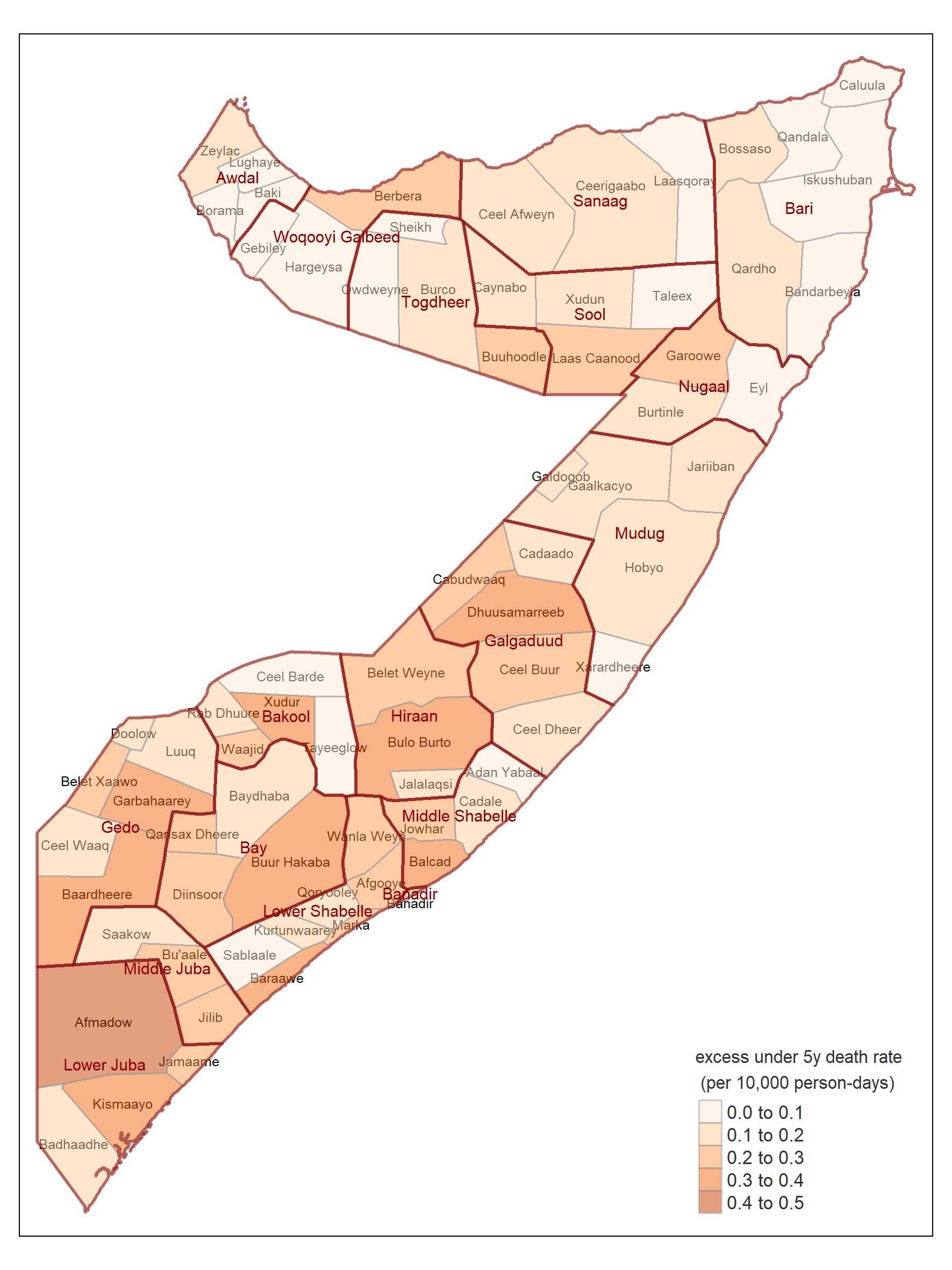


Fig L. Best estimate of excess under 5 years death rate among all age groups, by district. Map created in R software using basemap shapefile provided by the United Nations (<https://data.humdata.org/dataset/cod-ab-som>). Data license: Creative Commons Attribution (CC BY-IGO) (<https://data.humdata.org/faqs/licenses>).

## Trends in terms of trade indicators


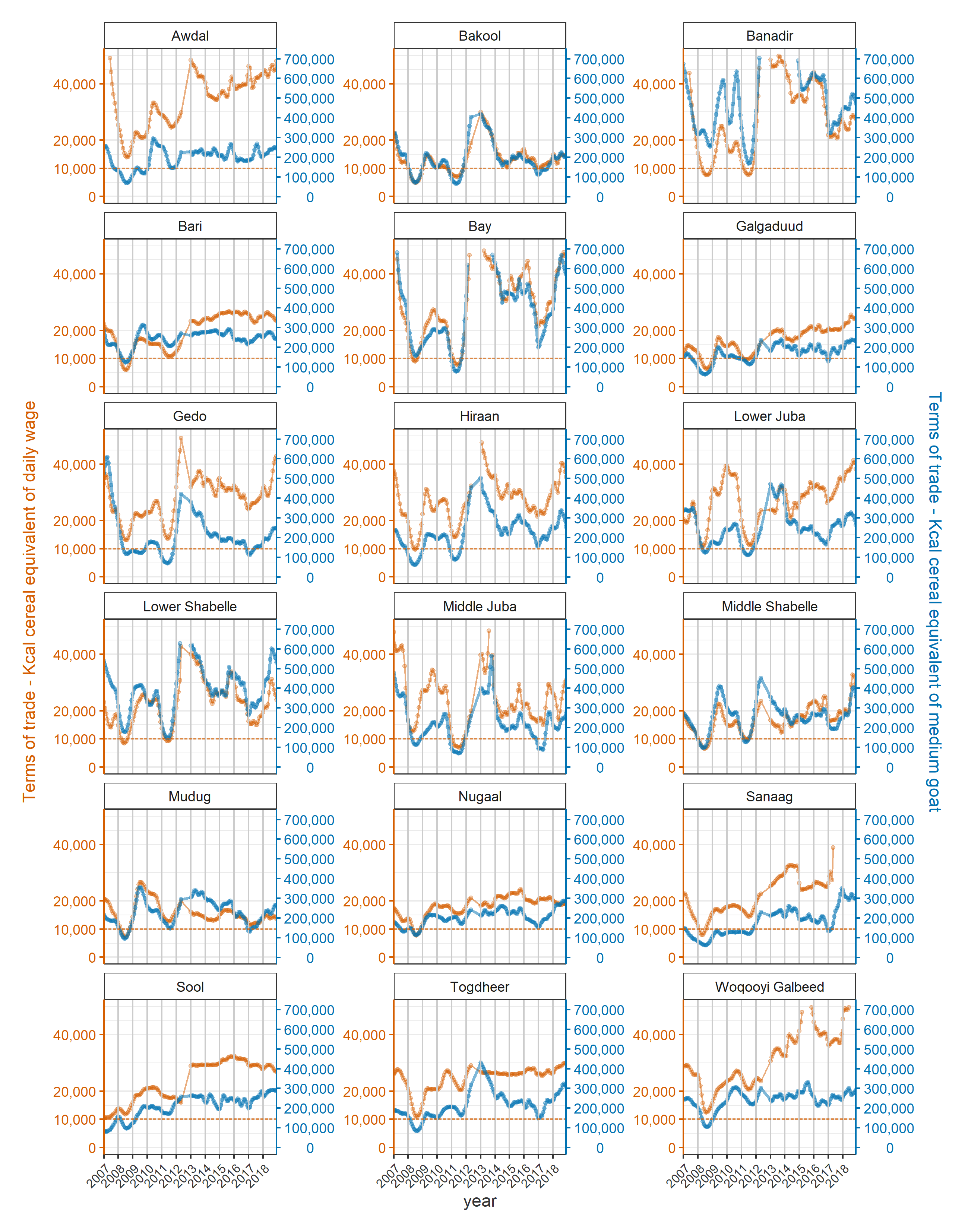


Fig M. Trends in terms of trade (daily wage vs. cereal, medium-quality goat vs. cereal), by region.

## Sensitivity analyses


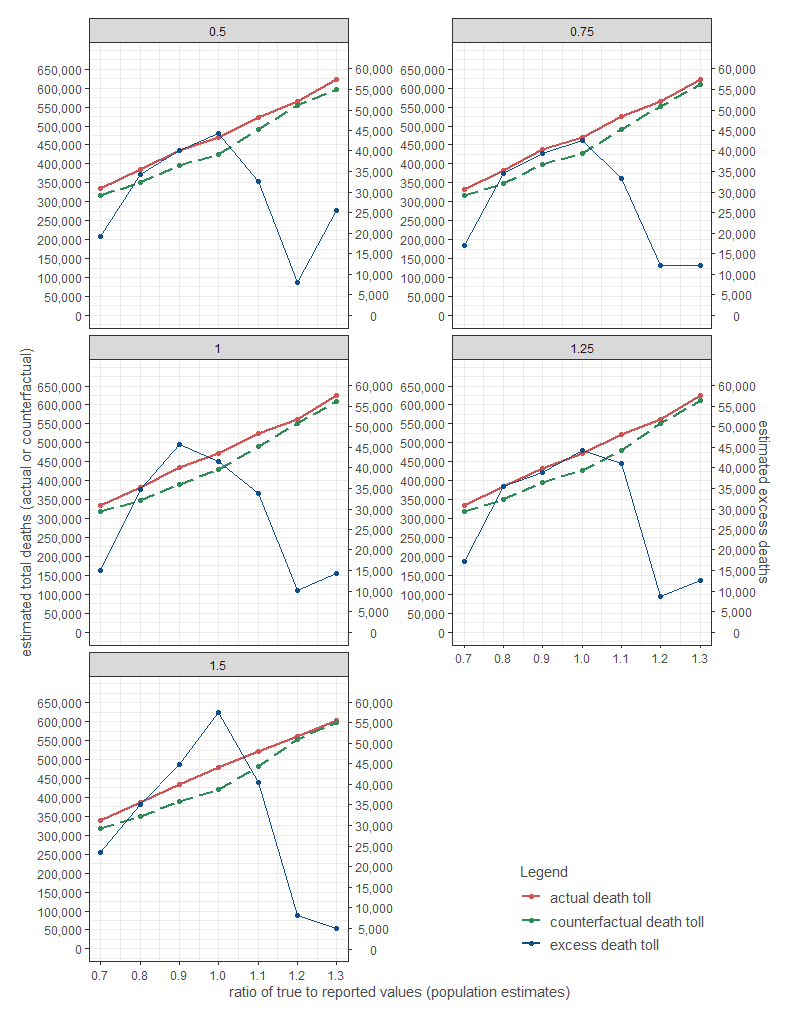


Fig N. Estimated actual, counterfactual (left axis) and excess (right axis) death toll for all age groups, by sensitivity value of the ratio of true to reported population estimates. Each panel presents results for different sensitivity values of the true number of internal displacements (as a ratio to the observed/reported value). Only the most likely counterfactual scenario is presented.


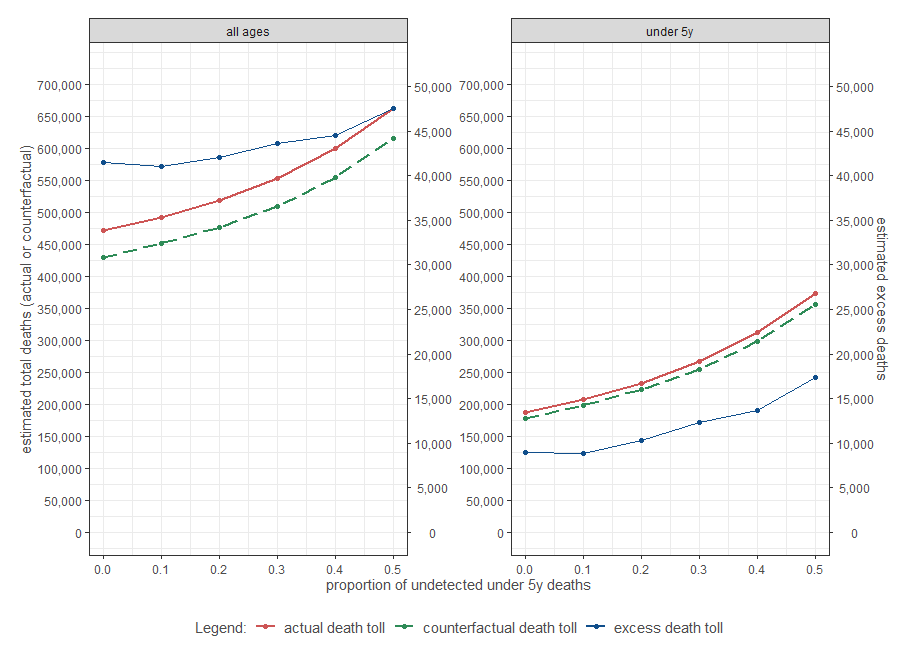


Fig O. Estimated actual, counterfactual (left axis) and excess (right axis) death toll for all age groups and children under 5y, by sensitivity value of the ratio of true to reported U5DR. Only the most likely counterfactual scenario is presented.
