# Supplementary material for: Drought, armed conflict and population mortality in Somalia, 2014–2018: A statistical analysis
Source: PLOS Glob Public Health. 2023 Apr 12;3(4):e0001136. doi: 10.1371/journal.pgph.0001136 (PMC10096495; doi:10.1371/journal.pgph.0001136)
Supplement: Attachment — Submitted filename: warsame_somalia_mortality_paper_rebuttal1.docx [file pgph.0001136.s002.docx]

Att.:	Editor, PloS Global Public Health

RE:	PGPH-D-22-01475

Drought, armed conflict and population mortality in Somalia, 2014-2018: a statistical analysis

3 December 2022

Dear Editor

Thank you for sharing our paper’s peer review outcome. Kindly see below point-by-point responses to the review:

**Journal Requirements:**

1. Please amend your detailed Financial Disclosure statement. This is published with the article. It must therefore be completed in full sentences and contain the exact wording you wish to be published.

a) State the initials, alongside each funding source, of each author to receive each grant. For example: "This work was supported by the National Institutes of Health (####### to AM; ###### to CJ) and the National Science Foundation (###### to AM)."

b) State what role the funders took in the study. If the funders had no role in your study, please state: “The funders had no role in study design, data collection and analysis, decision to publish, or preparation of the manuscript.”

*A: Done.*

2. Please update your online Competing Interests statement. If you have no competing interests to declare, please state: “The authors have declared that no competing interests exist.”

*A: Done.*

3. Please provide separate figure files in .tif or .eps format only and ensure that all files are under our size limit of 10MB.

*A: Done.*

4. We noticed that you used “unpublished” / “unpublished data” in the manuscript. We do not allow these references, as the PLOS data access policy requires that all data be either published with the manuscript or made available in a publicly accessible database. Please amend the supplementary material to include the referenced data or remove the references.

*A: What we meant is that the data were non-public at the time of acquisition, i.e. they weren’t already uploaded to a website or publicly accessible platform. We have changed ‘unpublished’ to ‘non-public’. ALL of the data, including these non-public datasets, are uploaded to the github repository for this study, as listed in the data availability statement accompanying the submission.*

5. Potential Copyright Issues:

Fig S4 and Fig S5: please (a) provide a direct link to the base layer of the map (i.e., the country or region border shape) and ensure this is also included in the figure legend; and (b) provide a link to the terms of use / license information for the base layer image or shapefile. We cannot publish proprietary or copyrighted maps (e.g. Google Maps, Mapquest) and the terms of use for your map base layer must be compatible with our CC-BY 4.0 license.

*A: Done.*

**Reviewers' comments:**

Under 'study design', I suggest adding in the github repository (https://github.com/francescochecchi/mortality_small_area_estimation) rather than only referencing the index paper for greater transparency.

*A: Done.*

I think Figure S1 is an important figure because it shows the distribution of data available per district, which aid interpretation of estimates described later in this manuscript. Please put this figure as a main figure in the manuscript. Figure S1 should have a scale for the colour. I believe the right Y-axis is the region in Somalia - please add the axis label.

*A: If we understand correctly, we are already at the limit in terms of figures allowed in the main manuscript, so we probably need to keep this figure in the appendix. We have improved the caption.*

Under 'ground mortality data', the authors state that there were 104 surveys excluded. However, the numbers of subsequent surveys excluded do not add up (i.e. 87 + 6). Please double check the numbers and provide an accurate description of the data sources. Perhaps just adding in the 11 from Figure 3 would do.

*A: The manuscript text has been amended to reflect Figure 3.*

Please consider combining figures 2 & 3 to make space for Figure S1.

*A: These seem like very different figures, and they also relate to different sections in the manuscript – not sure how we could combine them into one. Maybe the Editor can advise.*

'We used manual imputation and moderate spline smoothing to remove missingness and minimise outliers'. - I don't understand what this means. Please describe the imputation procedure in more detail and how splines were used to minimise outliers e.g. how did you define an appropriate spline function.

*A: Added more detail to the corresponding text.*

Predictive models - please provide details about how the model selection process took place. For example, it would be helpful to understand what metrics were used to decide on predictive accuracy. Also, please specify what comparable predictive accuracy means in the context of choosing a model. If possible, specifying the range of 'accuracies' within the top 20% best-fitting alternatives would be helpful to the readers. The calibration plots are informative but I cannot understand how you compared different predictive models.

*A: Added a bit more detail under this section of the methods. Agree this section was poorly written. We have also referred to the Checchi et al. Population Health Metrics paper that provides a lot more detail on this step.*

There are many reasons against a 80/20 splitting for internal validation. Given you have performed 10-fold cross validation, I wonder if you can simply remove the the 80/20 split. To confirm, were the models developed on the 80% data available?

*A: Sorry, this was poorly explained. No, models were developed on all the data and cross-validation was used as the main validation means (the text in the paper was carried over from previous iterations of the study). However, we also fit the model on an 80% training sample and predicted on the remaining 20%, just as a second validation check.*

In the sensitivity analyses, please describe what level of augmentation carried out. It is difficult to understand how this process happened with the current description.

*A: Thank you, we have tried to improve this section of the methods.*

Given the datasets are either in the public domain or shared in fully anonymised format, could the analytic script be shared on github or similar platform for greatest transparency?

*A: The github repository stated in the data sharing statement (and now in the main text) does contain both all the datasets and all the R scripts necessary to replicate the analysis in full.*

Under 'predictive model' section, I do not understand how Table 4 is helpful. The rationale for these models are for estimating mortality. I would find presenting the poisson regression formulae more useful, but probably as supplement.

*A: It’s helpful in our opinion because (i) the coefficients give some idea of the biological plausibility of the predictive model, and (ii) the predictor variables are not just used to predict mortality under observed conditions – they are also varied based on different counterfactual assumptions so as to predict baseline (non-crisis) mortality, so we think it’s important that the reader has transparency on how varying each of these predictors will affect the predictions.*

Again, I think S4 & S5 are very helpful figures. Could these be combined into one main figure? Ideally there would be a figure for U5DR as well.

*A: Not sure how they could be combined into a single, readable map. We do have a map of U5DR as well, but it’s predictably very similar to CDR and we don’t think it adds much information.*

Figure 5 needs some figure legends and captions.

*A: We have improved the caption, which was misleading.*

In the discussion, there is a section discussing the finding on the association between trade and mortality. This is not described in the body of the manuscript. The reference to 'the 2010-2012' study is confusing as there is no citation provided. If this is deemed important in the context of the paper, please separately report this finding in the results section, as well as describing the method to draw on your results. Otherwise, please consider removing this from the discussion.

*A: We have re-cited the 2010-2012 study. We think it’s reasonable to discuss this important difference between this study and a comparable study of the previous major drought in Somalia, by presenting some information in the main text and directing the reader to the Appendix. Again, adding another Figure to the main text doesn’t seem possible.*

Figure 6, i.e. new findings, should be presented in the results section and not in the discussion. The correlation described should be specified in the methods section - how was this done and what metric did you use. This is a very important figure because much of your conclusion is drawn from this figure. So clear explanation on the methods and the results would greatly improve the interpretation of this figure.

*A: Moved from discussion to results. We didn’t use any correlation metric, as we didn’t think such a statistic would have a meaningful interpretation. Rather, it seems visually apparent looking at Figure 6 that there is such a correlation.*

Throughout the text, please cite the specific figure/table in the supplement rather than citing S1 Appendix. There are also some abbreviations in the figures, so please provide figure legends that specify these abbreviations.

*A: Done.*

Best wishes

Francesco Checchi

[On behalf of co-authors]
