# Supplementary material for: Drought, armed conflict and population mortality in Somalia, 2014–2018: A statistical analysis
Source: PLOS Glob Public Health. 2023 Apr 12;3(4):e0001136. doi: 10.1371/journal.pgph.0001136 (PMC10096495; doi:10.1371/journal.pgph.0001136)
Supplement: Attachment — Submitted filename: warsame_somalia_mortality_paper_rebuttal2.docx [file pgph.0001136.s003.docx]

Att.:	Editor, PloS Global Public Health

RE:	PGPH-D-22-01475

Drought, armed conflict and population mortality in Somalia, 2014-2018: a statistical analysis

20 March 2023

Dear Editor

Thank you for sharing our paper’s peer review outcome. As far as we could see there was only one outstanding reviewer comment requiring revision (see below). Apart from this, we would be delighted if you could include our paper in the **Climate Change and Human Health series**, as suggested in your review outcome letter.

**Comments to the Author**

6. Review Comments to the Author

Please provide the Supplementary map Figure on the U5DR (although similar to the CDD) to retain equal attention to both estimates.

*A: Done. We have reuploaded the supplementary file featuring these additional figures.*

Best wishes

Francesco Checchi

[On behalf of co-authors]
